# Microbial Community, Fermentation Quality, and *in vitro* Degradability of Ensiling *Caragana* With Lactic Acid Bacteria and Rice Bran

**DOI:** 10.3389/fmicb.2022.804429

**Published:** 2022-05-31

**Authors:** Jingtao You, Huan Zhang, Hongfu Zhu, Yanlin Xue, Yimin Cai, Guijie Zhang

**Affiliations:** ^1^Department of Animal Science, Ningxia University, Yinchuan, China; ^2^Inner Mongolia Key Laboratory of Microbial Ecology of Silage, Inner Mongolia Engineering Research Center of Development and Utilization of Microbial Resources in Silage, Inner Mongolia Academy of Agriculture and Animal Husbandry Science, Hohhot, China; ^3^Japan International Research Center for Agricultural Science (JIRCAS), Tsukuba, Japan

**Keywords:** *Caragana* silage, fermentation quality, conserved forage, microbial population, rice bran, *Lactobacillus*

## Abstract

This study aimed to assess the effects of microbial inoculants and growth stage on fermentation quality, microbial community, and *in vitro* degradability of *Caragana* silage from different varieties. *Caragana intermedia* (CI) and *Caragana korshinskii* (CK) harvested at the budding (BU) and blooming (BL) stages were used as raw materials to prepare silage, respectively. The silages at each growth stage were treated for ensiling alone (control), with 5% rice bran (RB), a combination of RB with commercial *Lactobacillus plantarum* (RB + LP), and a combination of RB with a selected strain *Lactobacillus plantarum* L694 (RB + L694). The results showed that the crude protein (CP) content of CI was higher than that of CK, and delay in harvest resulted in greater CP content in *Caragana* at BL stage. After 60 days of fermentation, the concentrations of lactic acid (LA) in the RB + L694 treatments were higher than those in control treatments (*p* < 0.05), while the pH, concentrations of NH_3_-N, neutral detergent fiber with the addition of α-amylase (aNDF) were lower than those in control treatments (*p* < 0.05). RB + L694 treatments could decrease acid detergent fiber (ADF) content except in CIBL. In CK silages, adding RB + L694 could reduce bacterial diversity and richness (*p* < 0.05). Compared with the control, RB + L694 treatment contained higher *Lactobacillus* and *Enterobacter* (*p* < 0.05). *In vitro* NDF and DM degradability (IVNDFD and IVDMD) was mostly affected by growth period, and additive RB + l694 treatment had higher IVDMD and lower IVNDFD than other treatments (*p* < 0.05). Consequently, the varieties, growth stages, and additives could influence the fermentation process, while the blooming stage should be selected in both *Caragana*. Furthermore, the results showed that RB and *L. plantarum* could exert a positive effect on fermentation quality of *Caragana* silage by shifting bacterial community composition, and RB + L694 treatments outperformed other additives.

## Introduction

*Caragana* is a legume shrub that grows well in arid and semi-arid soils. It can be widely used to control desertification and provide ecological protection (Zhang et al., 2015). However, the overgrowth of *Caragana* may reduce the diversity of plant species and affect the ecological environment (Yang and Liu, 2019). Therefore, it is necessary to manage and effectively use *Caragana* to reduce species competition and enhance the stability of the ecosystem.

*Caragana intermedia* (CI) and *Caragana korshinskii* (CK) are the common cultivars in some arid regions of the world, including China. Both shrubs belong to the legume *Caragana* genus, and the fresh branches and leaves contain high-protein content and nutritional value, which can contribute to livestock production (Wang et al., 2021). Generally, the nutrient and palatability of *Caragana* are easily affected by the growth stage and the processing methods (Wang et al., 2020). Woody plants can accumulate large amounts of biomass, but the harvesting time is seasonally restricted. The natural drying process will cause nutrient loss, increase the lignin content, and reduce palatability. Therefore, silage is considered an alternative preparation and storage method that can be used as a convenient way to preserve woody forage and alleviate the problem of feed shortage in arid areas (Cai et al., 2020).

Generally, due to the low fermentation substrate of legumes, it is difficult to prepare woody plants naturally for obtaining high-quality silage (Cai et al., 2020). Ensiling shrub with lactic acid bacteria (LAB) and high sugar agricultural by-products may be an effective way to solve the fermentation problem. As a woody plant, *Broussonetia papyrifera* has high-protein content and nutritional value, which is similar to *Caragana*. Recent studies have shown that inoculated with LAB and molasses can improve the fermentation quality of *Broussonetia papyrifera* silage, change the microbial community structure, and reduce the number of harmful microorganisms (Basso et al., 2018; Du et al., 2021). Rice bran is refined to produce white rice and is also a cheap livestock feed (Shi et al., 2015). The previous study has shown that rice bran contains sugar, which can promote the growth of LAB and accelerate the fermentation of LA (Ono et al., 2014; He et al., 2020).

During ensiling, a regular succession of microbial communities occurs in the aerobic and anaerobic stages (Tao et al., 2020). For better understanding the fermentation process of ensiled, it is necessary to profile the bacterial communities (Dong et al., 2020; Ren et al., 2021). Recently, next-generation sequencing (NGS) has been widely used to study microbial communities in silage (Li et al., 2015; Zhang et al., 2017). The 16S rRNA (SSU rRNA) gene is regarded as the most widely used biomarker because it is present in the genomes of all bacteria (Grutzke et al., 2019).

The limitations of legume silage include low content of water-soluble carbohydrates (WSC) and high buffering capacity, which can result in a poor fermentation quality and nutrient loss of silage (Nkosi et al., 2016). This issue may be addressed by adding LAB and rice bran to increase WSC content and improve fermentability. Aerobic stability is an index to evaluate the difficulty of aerobic deterioration of silage. Poor aerobic stability caused dry matter (DM) and economic losses and decreased the nutrition of silages (Ferrero et al., 2019).

We hypothesized that the microbial community, fermentation quality, and *in vitro* degradability of *Caragana* silage could be improved by adding LAB and rice bran. Therefore, the purpose of this study is to investigate the microbial community, silage fermentation, aerobic stability, and *in vitro* degradability of *Caragana* prepared with LAB and rice bran.

## Materials and Methods

### Materials and Silage Making

*Caragana intermedia* (CI) and *Caragana korshinskii* (CK) used in this experiment were planted in 2014 in an experiment field (107° E, 37° N, Yanchi, China), which was harvested at the budding stage (BU) on April 17, 2019, and the blooming stage (BL) on June 25, 2019. Both fresh shrubs were harvested from three randomly selected locations within the field as three repetitions, using hand clippers (400–700, Jingmei Linglang Trading Co., Ltd., Shenzhen, China) and leaving a stubble of 10 cm above ground.

After the cutting, the raw shrub was broken and kneaded into a slice less than 1 mm, using a kneading machine (RC-400, QuFuZhiZao Conveyor Co., Ltd., Shandong, China). The fresh shrubs were adjusted to a DM content of about 40% by adding water and then treated without additive (control) and with 5% rice bran (RB), 5% RB plus commercial LAB (*Lactobacillus plantarum* LP) inoculant (RB + LP), and 5% RB plus a selected strain *Lactobacillus plantarum* L694 (RB + L694).

The proportions of crude protein, crude fiber, crude fat, and water-soluble carbohydrates in RB are 12.8, 5.7, 16.5, and 8.58% DM, respectively, which were derived from a by-product of rice production, provided by a local feed company, and 5% of the fresh material was used for mixing with shrub samples. Strain L694 was isolated from high-moisture corn silage, which could grow under low pH conditions and produce more LA in the silage environment, provided by Sichuan Agricultural University. Strain L694 was cultured in MRS agar (HB0384, Hope Bio-Technology Co., Ltd., Qingdao, China) for 24 h for silage preparation. The plate counting method was used to determine the number of viable bacteria. The final addition amount was 3 × 10^5^ colony-forming unit (CFU)/g of fresh weight and mixed evenly with *Caragana* sample. The commercial LAB was mixed with sterile water at an added amount of 3 × 10^5^ CFU/g of fresh weight and uniformly sprayed on the *Caragana* sample. Silages were prepared with a laboratory-scale fermentation system with bag silos, which weigh 500 g of fresh material and then pack it into a polyethylene bag (270 mm × 300 mm; Embossed Food saver bag Co., Ltd., Chengdu, China) with a one-way exhaust valve. A vacuum packaging machine (DZ-400, Shandong Zhucheng Yizhong Machinery Co., Ltd., Zhucheng, China) was used to vacuum compress and seal the bag. There were 48 bag silos with three replicates of silage per treatment stored at room temperature (24°C–26°C) for 60 days.

### Quality Analysis of Silage

The bag silos were opened on Day 60 after ensiling. 10 g silage samples were diluted with 90 ml of distilled water, filtered with four layers of cheesecloth, and stored in a refrigerator at 4 °C for 24 h. The supernatant was then measured for pH using a calibrated pH meter (PHS-3G, Mettler Toledo, Zurich, Switzerland). Before analyzing ammonia nitrogen and organic acid, a subsample of the supernatant was centrifuged at 2,500 rpm for 10 min and passed through a 0.22-μm microporous filter. The ammonia nitrogen content was analyzed according to the procedure of Broderick and Kang (1980). Organic acid was analyzed using high-performance liquid chromatography (HPLC) (KC-811 column, Shodex; Shimadzu: Japan; oven temperature 50°C; flow rate: 1 ml/min; SPD: 210 nm) as described by Tian et al. (2014).

The materials and silage samples of CI and CK were dried in a forced air oven at 105°C for 15 min and turn down to 65 °C immediately. After drying for 48 h, it was milled and passed through a 1-mm screen and then heated to 105°C until their weight was constant for the analyzed dry matter (DM). The crude protein (CP) was calculated by multiplying 6.25 with the content of nitrogen (N), which was determined using the Kjeldahl apparatus (K-360, BUCHI laboratory equipment trade Co., Ltd., Shanghai, China). NH_3_-N content was determined by the phenol-sodium hypochlorite colorimetric method (Sekerka and Lechner, 1974).

Neutral detergent fiber with the addition of α-amylase (aNDF), acid detergent fiber in organic matter (ADF), and acid detergent lignin (ADL) were determined according to the methods of Van Soest et al. (1991) using an ANKOM A2000i fiber analyzer (A2000i, ANKOM Technology, New York, United States). Water-soluble carbohydrates were determined by anthrone-sulfuric acid colorimetry (Murphy, 1958).

### Aerobic Stability Analysis

After 60 d of ensiling, the silages with three replicates per treatment were placed in a new polyethylene bag (270 mm × 300 mm; Embossed Food saver bag; Changyang, Chengdu, China) and stored at room temperature (24∼26°C), and each bag was portioned 500 g silages. A HOBO Pendant Temperature Data Logger (Onset Ltd., Massachusetts, United States) was put and punctured holes in the geometric center of the polyethylene bag that recorded the temperature every 30 min for each bag. Another temperature data logger was placed in the room to record room temperature. The silage was considered to have deteriorated when the silage temperature was 2°C more than the room temperature.

### Bacterial Community of *Caragana*

The microbial total DNA extraction was performed according to the method of Zheng et al. (2017). Twenty grams of sample was collected, mixed with 80 ml of sterile water, and stirred at 120 rpm and 4°C for 2 h. The samples were filtered through two layers of sterile gauze and then centrifuged at 10,000 × *g* for 15 min at 4°C.

The DNA of silage was extracted by the FastDNA SPIN for Soil Kit (MP Biomedicals, Solon, United States). The final DNA concentration and purification were determined by NanoDrop 2000 UV-vis spectrophotometer (Thermo Fisher Scientific, Wilmington, United States), and DNA quality was checked by 1% agarose gel electrophoresis. The V3–V4 hypervariable regions of the bacteria 16S rRNA gene were amplified with primers 338F (5′- ACTCCTACGGGAGGCAGCAG-3′) and 806R (5′-GGACTACHVGGGTWTCTAAT-3′) by thermocycler PCR system (GeneAmp 9700, ABI, United States).

The PCR reactions were conducted using the following program: 3 min of denaturation at 95°C, 27 cycles of 30 s at 95°C, 30 s for annealing at 55°C, and 45 s for elongation at 72°C, and a final extension at 72°C for 10 min. PCR reactions were performed in triplicate 20 μL mixture containing 4 μL of 5 × FastPfu Buffer, 2 μL of 2.5 mM dNTPs, 0.8 μL of each primer (5 μM), 0.4 μL of FastPfu Polymerase, and 10 ng of template DNA. The resulted PCR products were extracted from a 2% agarose gel and further purified using the AxyPrep DNA Gel Extraction Kit (Axygen Biosciences, Union City, CA, United States) and quantified using QuantiFluor™-ST (Promega, United States) according to the manufacturer’s protocol.

The reads less than 50 bp were discarding to obtained clean reads, which were clustered at the similarity of 97% into operational taxonomic units (OTUs) to investigate species diversity of all the samples by Uparse software. The OTUs were annotated by the Silva (SSU123) to obtain the composition of each sample. Alpha diversity analysis was performed using the Mothur software platform. Principal component analysis (PCA) was performed and plotted in R software (version 3.5.1).

### *In vitro* Digestibility

The *in vitro* digestibility of dry matter (IVDMD) and NDF (IVNDFD) were measured according to Tian et al. (2014). Dried and ground silage samples (1 g, milled through a 1.0 mm screen) were incubated in the pepsin-hydrochloric acid solution for 16 h, followed by hydrolysis with a pH 4.6 cellulase–acetate buffer for 48 h, then inactivated at 90°C for 30 min, and washed with distilled water. After dried at 105°C to constant weight, the residue was weighted, and IVDMD was calculated. The content of NDF in the residue of enzymatic hydrolysis was determined, and IVNDFD was then calculated.

### Statistical Analysis

Data on chemical composition, fermentation characteristics, alpha diversity, and *in vitro* degradability were analyzed via a 2 × 4 × 4 factorial design according to the model: Yijk = μ + Vi + Gj + Ak + (V × G)ij + (V × A)ik + (G × A)jk + (V × G × A)ijk + eijk, where Yij = observation; μ = overall mean; Vi = effect of varieties (i = 1,2); Gj = effect of growth stages (j = 1, 2); Ak = effect of additives (k = 1, 2, 3, 4); (V × G)ij = effect of interaction between varieties and growth stages; (V × A)ik = effect of interaction between varieties and additives; (G × A)jk = effect of interaction between growth stages and additives; (V × G × A)ijk = effect of interaction between varieties, stages, and additives, and eijk was the residual error. The model includes two varieties, two growth stages, four additives, and their interactions. Tukey’s HSD was used for multiple comparisons with difference declared significant at *p* < 0.05.

## Results

### Chemical Composition of Fresh *Caragana* Before Ensiling

Chemical composition of fresh *Caragana* before ensiling is shown in Table 1. The NDF content was affected by varieties, growth stage, and their interactions (*p* < 0.05). The CK had higher NDF and ADF than CI (*p* < 0.05), whereas CI contained higher CP than CK (*p* < 0.05). The fiber, CP, and WSC concentrations at the blooming stage were higher than those at the budding stage (*P* < 0.05).

**TABLE 1:** Chemical composition of fresh *Caragana* before ensiling (*n* = 3).

Items^1^	Materials^2^				SEM^3^	*P*-value^4^		
	CIBU	CIBL	CKBU	CKBL		V	G	V × G
DM (%)	41.04	40.35	40.94	41.13	0.24	NS	NS	NS
CP (% DM)	12.41^b^	14.07^a^	10.36^d^	11.48^c^	0.10	**	**	NS
NDF (% DM)	70.01^c^	71.19^c^	73.36^b^	77.07^a^	0.32	**	**	**
ADF (% DM)	51.30^c^	54.58^b^	57.76^ab^	59.56^a^	0.71	**	**	NS
ADL (% DM)	16.59^b^	20.63^a^	18.96^a^	19.54^a^	0.44	NS	**	**
WSC (% DM)	1.98^b^	3.11^ab^	2.30^b^	4.25^a^	0.36	NS	**	NS

*^1^DM, dry matter; CP, crude protein; NDF, neutral detergent fiber; ADF, acid detergent fiber; ADL, acid detergent lignin; WSC, water-soluble carbohydrate.*

*^2^CI, Caragana intermedia; CK, Caragana korshinskii; BU, budding stage**;** BL, blooming stage.*

*^3^SEM, standard error of the means.*

*^4^V, varieties; G, growth stage; NS, no significant.*

**, p < 0.01.

*^a–d^Means of inoculation treatments within a row with different superscripts differ (p < 0.05).*

### Fermentation Characteristics of *Caragana* Silage

**FIGURE 1:**
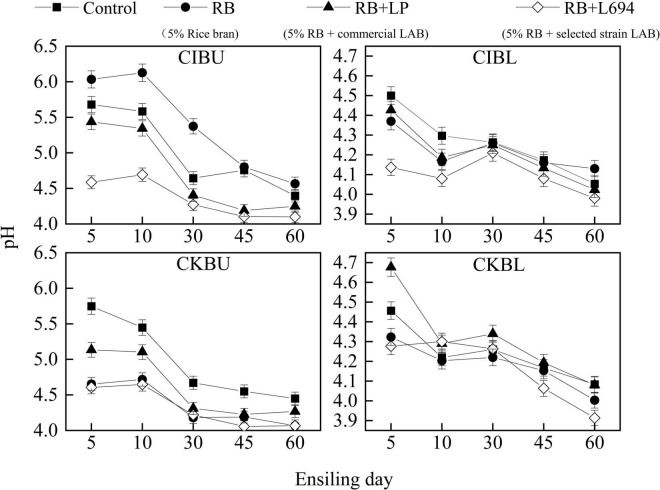
pH dynamics of *Caragana intermedia* (CI) and *Caragana korshinskii* (CK) silages at the budding (BU) and blooming stages (BL) during ensiling. RB, 5% rice bran; LP, commercial inoculant strain *Lactobacillus plantarum*; L694, a selected strain *Lactobacillus* (*n* = 3).

The fermentative changes in pH of silage are shown in Figure 1. The pH of CIBU silages treated with RB + L694 was lower than other treatments in the whole silage process (*p* < 0.05), which was 4.58 on Day 5 decreased to 4.1 at the end of ensiling. Although the pH of RB treatment group was not different from that of control group on Day 45 (*p* > 0.05), the pH value for the rest of the time was significantly higher than that of control group (*p* < 0.05). In CIBL, the initial pH value of RB + L694 treatment was 4.13, and the final pH value decreased to 3.98; furthermore, the pH of the whole silage process was significantly lower than other treatment groups (*p* < 0.05). In CKBU, pH of the whole ensiling stage in all treatment groups was lower than that in control group (*p* < 0.05). On Day 30, pH in all treatment groups decreased to 4.5 and remained at the lower level until Day 60; RB + L694 and RB treatments had the lowest final pH, which was 4.06 and 4.07. In CKBL, the RB + L694 treatment group had the lowest pH (*p* < 0.05) on Day 60, which was 3.91. In addition, during ensiling treated with CIBL and CKBL silages, pH further reduced was observed after 30 days, whereas treated with CIBU and CKBU silages pH was unchanged.

**FIGURE 2:**
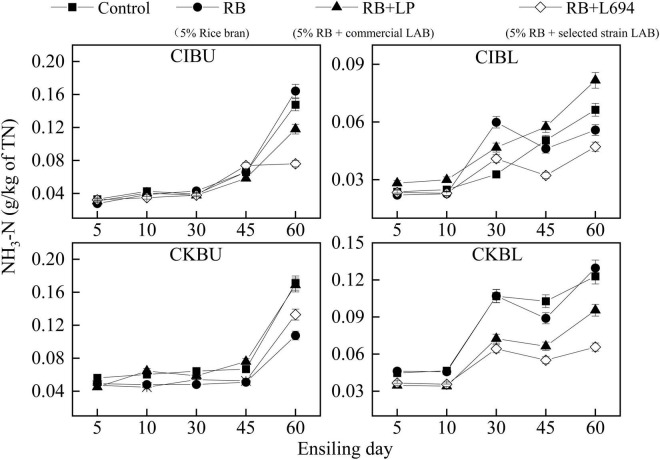
NH_3_-N dynamics of *Caragana intermedia* (CI) and *Caragana korshinskii* (CK) silages at the budding (BU) and blooming stages (BL) during ensiling. RB, 5% rice bran; LP, commercial inoculant strain *Lactobacillus plantarum*; L694, a selected strain *Lactobacillus* (*n* = 3).

The fermentative changes in NH_3_-N of silage are shown in Figure 2. The initial NH_3_-N content in CIBU was all below 0.04 g/kg of total nitrogen (TN). On Day 60 of ensiling, the NH_3_-N of the RB + L694 treatment group was 0.076 g/kg TN, which was lower than that of the other treatments (*p* < 0.05). In CIBL, the NH_3_-N of the RB treatment group increased rapidly from Days 10 to 30, from 0.021 to 0.059 g/kg TN, which was higher than that of the control group (*p* < 0.05). The NH_3_-N of RB + L694 on Days 45 and 60 was lower than that of other treatments (*p* < 0.05), which was 0.032 and 0.047 g/kg TN, respectively. In CKBU, the increasing trend of NH_3_-N in different treatment groups was relatively slow in the first 45 days. While on Day 60, the NH_3_-N in RB treatment group and RB + L694 group was lower than that in the control group (*p* < 0.05), 0.10 and 0.13 g/kg TN, respectively. There was no difference between RB + LP treatment group and control group (*p* > 0.05). The CKBL group was similar to the CIBL group, with a slower increase on days 5–10. On Day 60, the NH_3_-N of RB + L694 was 0.06 g/kg TN, which was lower than that of other treatments (*p* < 0.05). In addition, the NH_3_-N contents tardily increased in the first 10–30 days during ensiling and then peaked after 60 days.

As shown in Table 2, the lactic acid (LA), acetic acid (AA), and LA/AA contents were mainly affected by interaction between varieties, growth stage, and additives (*p* < 0.01). LA content in additive treatments was significantly higher than control group (*p* < 0.05). RB + L694 treatments significantly reduced AA content and increased LA/AA content (*p* < 0.05), compared with other treatment groups. The varieties × growth stage and varieties × additives had effects on propionic acid (PA) content. In CIBU, additives had no significant effect (*p* > 0.05).

**TABLE 2:** Fermentation quality, IVDMD, and IVDFD of *Caragana* silages prepared without and with RB and LAB at different growth stages (*n* = 3).

Item	Material^2^	*Caragana* silage treatmen^3^				SEM^4^	*P*-value^5,6^						
		Control	RB	RB + LP	RB + L694		V	G	A	V × G	V × A	G × A	V × G × A
Lactic acid (% of DM)	CIBU	7.32^C^ ^a^	9.47^B a^	8.88^B a^	10.66^A a^	0.34	**	**	**	**	**	NS	**
	CIBL	8.27^B a^	8.85^AB a^	9.81^A a^	10.19^A a^								
	CKBU	5.77^B b^	6.65^B b^	9.29^A a^	8.64^A b^								
	CKBL	6.86^B ab^	9.13^A a^	9.93^A a^	9.91^A a^								
Acetic acid (% of DM)	CIBU	2.13^A ab^	1.64^B c^	1.83^B b^	1.83^B b^	0.06	NS	NS	**	**	**	**	**
	CIBL	2.40^A a^	1.91^B b^	2.02^B a^	1.67^C c^								
	CKBU	1.99^B b^	2.23^A a^	2.07^AB a^	2.02^B a^								
	CKBL	2.12^A ab^	1.61^C c^	1.90^B ab^	1.84^B b^								
Propionic acid (% of DM)	CIBU	0.043^A a^	0.023^B b^	0.037^A a^	0.040^A b^	0.004	*	**	*	**	**	*	*
	CIBL	0.033^A b^	0.023^A b^	0.027^A a^	0.034^A b^								
	CKBU	0.047^ABa^	0.053^A a^	0.030^B a^	0.053^A a^								
	CKBL	0.020^C c^	0.036^A ab^	0.033^ABa^	0.027^BC^ ^b^								
LA/AA	CIBU	3.44^C a^	5.76^A a^	4.85^B ab^	5.83^A a^	0.19	**	**	**	**	**	NS	**
	CIBL	3.46^C a^	4.63^B b^	4.85^B ab^	6.09^A a^								
	CKBU	2.90^B a^	2.99^B c^	4.50^A b^	4.29^A b^								
	CKBL	3.24^B a^	5.68^A a^	5.23^A a^	5.41^A a^								
Aerobic stability (h)	CIBU	113.67^A a^	115.00^A a^	102.33^A a^	115.83^A a^	2.30	NS	**	**	**	**	**	**
	CIBL	36.67^D b^	41.17^C c^	65.17^B c^	70.50^A c^								
	CKBU	125.17^A a^	115.17^B a^	87.17^D b^	93.17^B b^								
	CKBL	36.00^D b^	70.33^B b^	56.50^C c^	89.83^A bc^								
*In vitro* digestibility^1^													
IVDMD (%DM)	CIBU	53.51^C b^	55.98^B c^	57.43^AB b^	59.25^A b^	0.86	**	**	**	**	**	**	NS
	CIBL	58.40^C a^	63.38^AB a^	62.55^B a^	66.54^A a^								
	CKBU	46.82^B c^	52.34^A d^	51.62^A c^	52.74^A d^								
	CKBL	46.83^D c^	59.61^A b^	53.92^C c^	56.49^B c^								
IVNDFD (%DM)	CIBU	16.87^A c^	16.74^A bc^	17.63^A b^	16.42^A b^	0.29	**	**	**	NS	NS	*	NS
	CIBL	16.76^A c^	15.78^B c^	16.06^AB c^	14.90^C c^								
	CKBU	19.80^A a^	18.85^AB a^	19.99^A a^	18.26^B a^								
	CKBL	18.62^A b^	17.05^B b^	17.64^B b^	17.77^B a^								

*^1^IVDMD, in vitro dry matter digestibility; IVNDFD, in vitro neutral detergent fiber digestibility.*

*^2^CI, Caragana intermedia; CK, Caragana korshinskii; BU, budding stage**;** BL, blooming stage.*

*^3^RB, 5% rice bran; RB + LP, 5% rice bran + commercial inoculant strain Lactobacillus plantarum; RB + L694, 5% rice bran + strain Lactobacillus 694.*

*^4^SEM, standard error of the means.*

*^5^V, varieties; G, growth stage; A, additives; V × G, interaction between varieties and growth stage; V × A, interaction between varieties and additives; G × A, interaction between growth stage and additives; V × G × A, interaction between varieties, growth stage, and additives.*

^6^NS, no significant, *p < 0.05; **p < 0.01.

*^A–D^Means of inoculation treatments within a row with different superscripts differ (p < 0.05).*

*^a–d^Means of variety and growth stage within a column with different superscripts differ (p < 0.05).*

The aerobic stability in CIBL and CKBL, and additive treatment group was significantly higher than that of control group (*p* < 0.05), and RB + L694 treatment group was the highest (*p* < 0.05). The use of additives in CKBU can reduce its aerobic stability (*p* < 0.05), while BU stage had higher aerobic stability than that of BL stage (*p* < 0.05).

### Alpha Diversity of *Caragana* Silage

As shown in Table 3, the alpha diversity changed with varieties, growth stage, and additives. All samples had relatively high coverage (> 0.99). Both Shannon index and Simpson index were affected interactively by variety, growth period, and additives (*P* < 0.01). The varieties and additives affected interactively the indexes of Ace and Chao1 (*P* < 0.01). In addition, the indexes of Shannon, Ace, and Chao1 in additive treatment were higher than those in control treatment (*P* < 0.01). The index of Simpson in additive treatment was lower than that in control treatment (*P* < 0.01).

**TABLE 3:** Analysis of alpha diversity of *Caragana* silage after ensiling (*n* = 3).

Items	Material	Fresh shrub	Treatment				SEM		*P*-value					
			Control	RB	RB + LP	RB + L694		V	G	A	V × G	V × A	G × A	V × G × A
Shannon	CIBU	1.91	0.91	2.06	1.64	1.47	0.069	**	**	**	*	**	*	**
	CIBL	3.15	1.59	2.72	1.66	2.23								
	CKBU	1.06	1.46	1.92	1.64	0.98								
	CKBL	2.32	1.04	0.84	3.10	0.17								
Simpson	CIBU	0.45	0.66	0.20	0.29	0.46	0.022	**	NS	**	*	**	*	**
	CIBL	0.16	0.38	0.19	0.55	0.24								
	CKBU	0.65	0.35	0.23	0.36	0.68								
	CKBL	0.35	0.53	0.70	0.08	0.95								
Ace	CIBU	490.44	137.60	258.07	206.11	259.26	8.584	NS	NS	**	**	**	*	NS
	CIBL	557.65	134.70	245.44	260.24	431.02								
	CKBU	598.49	223.85	308.87	191.37	161.39								
	CKBL	492.41	136.93	148.36	231.79	180.37								
Chao1	CIBU	486.87	130.79	202.06	201.16	261.07	8.409	**	NS	**	**	**	NS	NS
	CIBL	557.47	124.66	252.10	264.75	415.74								
	CKBU	494.67	168.78	296.79	172.35	162.48								
	CKBL	487.79	114.23	134.75	241.73	137.12								
Coverage	CIBU	0.9984	0.9993	0.9990	0.9993	0.9991	0.00	NS	**	**	NS	*	**	NS
	CIBL	0.9993	0.9995	0.9995	0.9996	0.9985								
	CKBU	0.9980	0.9992	0.9989	0.9992	0.9997								
	CKBL	0.9990	0.9995	0.9997	0.9996	0.9994								

BU, budding stage; BL, blooming stage; CI, C. Intermedia; CK, C. korshinskii; RB, 5% rice bran; RB + LP, 5% rice bran plus commercial inoculant strain Lactobacillus plantarum; RB + L694, 5% rice bran plus strain Lactobacillus 694; V, varieties; G, growth stage; A, additives; NS, no significant, *p < 0.05; **p < 0.01.

### Principal Component Analysis of *Caragana* Silage

**FIGURE 3:**
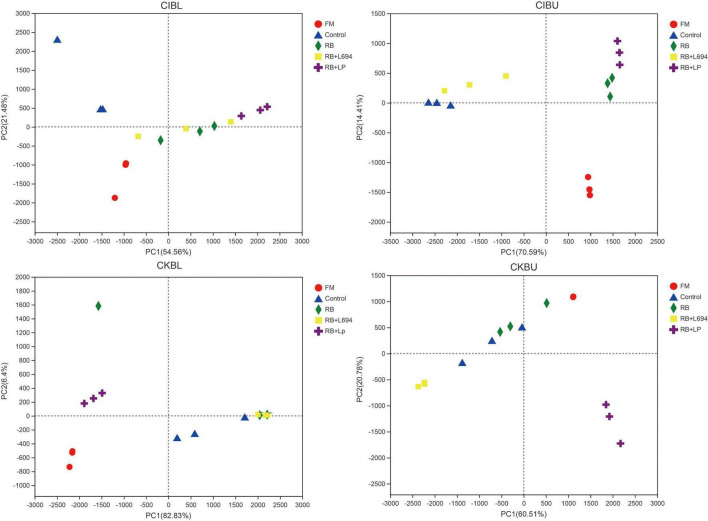
PCA analysis of OTU level for *Caragana intermedia* (CI) and *Caragana korshinskii* (CK) silages at the budding (BU) and blooming stages (BL) after 60 days of ensiling. RB, 5% rice bran; LP, commercial inoculant strain *Lactobacillus plantarum*; L694, a selected strain *Lactobacillus* (*n* = 3).

As shown in Figure 3, all fresh materials were grouped into a single category and were far away from the samples of other treatment groups. In CIBL, RB + LP silage aggregation was in the second quadrant, the distance within the group was relatively close, and the control group is clustered into one category. In CIBU, RB and RB + LP silages were clustered into one group, while control and RB + L694 silages were clustered into one group. The samples in the two groups were close to each other, and there was little difference in species diversity. In CKBL, RB + LP, and RB + L694 were clustered into two different separate groups, and one single sample in RB treatment was far away from the other two samples, and there were great differences in species diversity. Similar to CKBL, RB + LP, and RB + L694 silages in CKBU were grouped separately, while the distance between control and RB samples was large.

### Bacterial Composition Analysis of *Caragana* Silage

**FIGURE 4:**
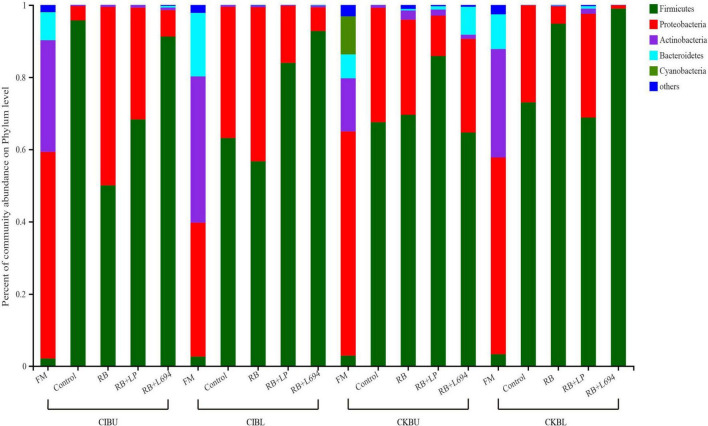
Bacterial communities and relative abundance of *Caragana* materials and their silages at phylum level (*n* = 3).

**FIGURE 5:**
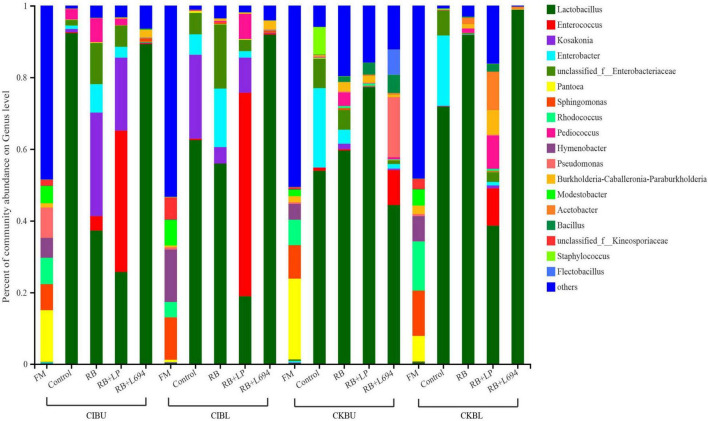
Bacterial communities and relative abundance of *Caragana* materials and their silages at genus level (*n* = 3).

**FIGURE 6:**
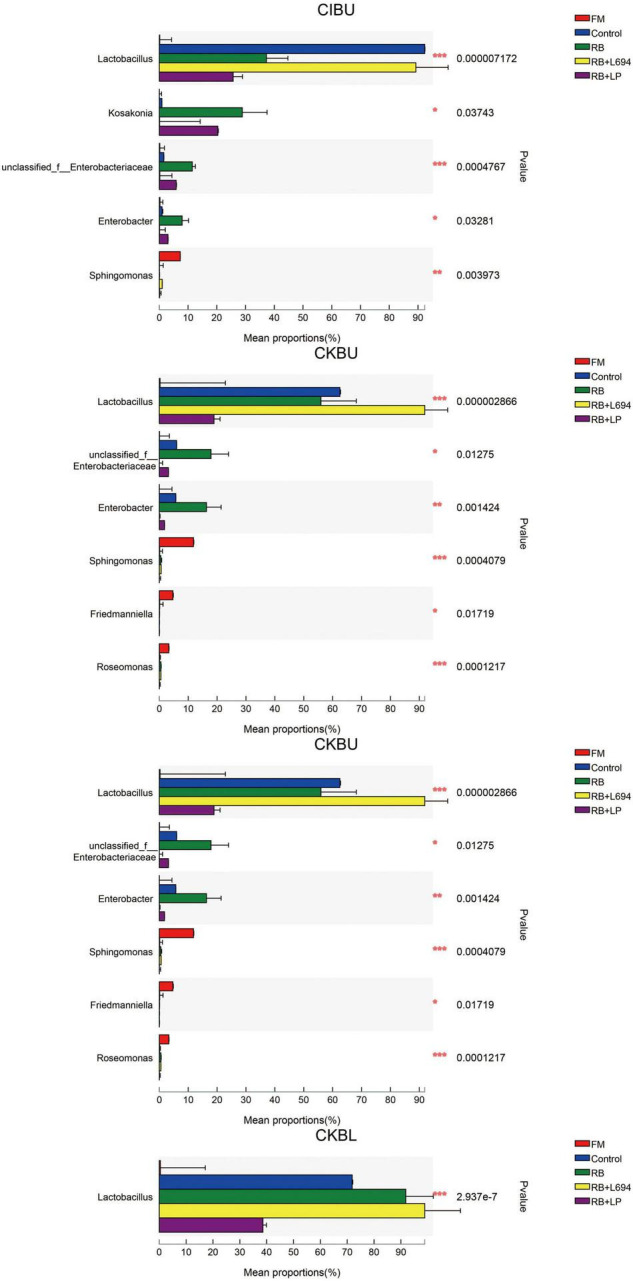
Mean proportions between bacterial communities and relative abundance of *Caragana* materials and their silages at phylum level and genus level (*n* = 3).

The fresh *Caragana* and silage had mainly five phyla and 18 genera (Figures 4–6). *Proteobacteria* were the main phylum in fresh *Caragana*, with 57.24% of abundance, while the most dominant phylum (*p* < 0.05) in *Caragana* silages was *Firmicutes* (50.09–98.99%). For CIBU, *Pantoea* were dominant in fresh *Caragana* (14.41%) (*p* < 0.05), and *Lactobacillus* was the most predominant epiphytic bacteria (*p* < 0.05) in RB + L694 and control silages, which was 92.34 and 89.26%, respectively. *Enterococcus* (39.44%) was the main bacteria in RB + LP silages. For CKBU, *Sphingomonas* (11.83%) and *Hymenobacter* (14.69%) were dominant in the fresh *Caragana*. The dominant bacteria of RB + L694 silage were *Lactobacillus*, with a relative abundance of 91.88%, significantly higher than that of the control group (*p* < 0.05). For CIBL, fresh *Caragana* has the most relative abundance (*p* < 0.05) of *Sphingomonas* (22.54%), same as CIBU silages. *Lactobacillus* (44.35%) was the predominant epiphytic bacteria in RB + L694, followed by *Pseudomonas* (16.75%) and *Enterococcus* (9.76%). *Lactobacillus* (77.24%) was dominant in RB + LP silage. For CKBL, *Rhodococcus* (13.72%) was dominant in the fresh *Caragana*. RB + L694 had the most relative abundance (*p* < 0.05) of *Lactobacillus*, which was 98.88%. In RB + LP silage, it also appeared *Enterococcus* (10.44%), *Acetobacter* (10.77%), and *Pediococcus* (9.24%). At the genus level (Figures 5, 6), the most dominant bacterial genus (*p* < 0.05) in the blooming stage silages was *Lactobacillus*, with more than 40% of abundance. However, *Enterococcus* was the most dominant genus (*p* < 0.05) in CI (9.44%) and CK (56.77%) which was treated with RB + LP, while varied microbes were observed in fresh *Caragana*.

### Chemical Composition of *Caragana* Silage

The chemical compositions of *Caragana* silage are shown in Table 4. The varieties, growth stage, varieties × growth stage, and varieties × additives had effects on CP content (*p* < 0.05), and additives did not affect the CP content, while influenced by the interaction of the three factors. The varieties, growth stage, additives, and their interactions affected the content of aNDF, ADF, ADL, and WSC content (*p* < 0.05). The concentrations of CP, fiber, and WSC of *Caragana* silage in the blooming stage (*p* < 0.05) increased, and the CP and fiber contents in *C. intermedia* silage were higher and lower than *C. korshinskii* silage, respectively. The RB and RB + L694 treatments effectively reduced the fiber content.

**TABLE 4:** Chemical composition of *Caragana* silages prepared without and with RB and LAB at different growth stages (*n* = 3).

Item	Material^1^	*Caragana* silage treatmen^2^				SEM^3^	*P*-value^4,5^						
		Control	RB	RB + LP	RB + L694		V	G	A	V × G	V × A	G × A	V × G × A
DM (%)	CIBU	40.04	39.48	40.58	40.36	0.68	NS	NS	NS	NS	NS	NS	NS
	CIBL	39.35	41.07	41.33	39.97								
	CKBU	39.94	40.50	39.96	39.47								
	CKBL	40.46	40.82	39.85	39.65								
CP (% of DM)	CIBU	11.70^A a^	11.85^A a^	11.38^A a^	11.45^A a^	0.22	**	**	NS	**	**	**	**
	CIBL	13.19^AB b^	14.11^A b^	12.57^B b^	13.71^A b^								
	CKBU	9.64^A c^	9.33^B c^	9.67^A b^	9.79^A b^								
	CKBL	9.92^AB c^	9.50^B d^	11.19^A c^	11.09^A c^								
aNDFom (% of DM)	CIBU	70.34^A b^	65.77^B a^	66.05^B c^	65.90^B b^	0.89	**	**	**	**	**	**	**
	CIBL	65.62^A c^	56.82^C b^	68.80^A ab^	61.20^B c^								
	CKBU	72.97^A ab^	66.24^C a^	69.99^B a^	69.05^B a^								
	CKBL	75.95^A a^	66.10^C a^	66.97^BC bc^	69.14^B a^								
ADFom (% of DM)	CIBU	54.58^A b^	50.92^B a^	49.66^B b^	49.19^B b^	0.76	**	**	**	**	**	**	**
	CIBL	46.73^B c^	42.79^C b^	51.57^A a^	45.56^BC c^								
	CKBU	56.18^A b^	51.08^C a^	54.83^AB a^	53.65^B a^								
	CKBL	59.76^A a^	52.40^B a^	51.93^B a^	52.82^B a^								
ADL (% of DM)	CIBU	20.68^A a^	17.90^B a^	15.59^C b^	13.73^C b^	0.45	**	**	**	*	**	**	**
	CIBL	15.13^AB c^	14.49^B c^	14.93^AB b^	15.54^A a^								
	CKBU	18.64^A b^	16.02^B b^	17.46^A a^	15.85^B a^								
	CKBL	18.75^A b^	16.44^B b^	18.56^A a^	16.56^B a^								
WSC (% of DM)	CIBU	0.33^C c^	0.68^B d^	1.23^A c^	1.34^A c^	0.08	**	**	**	**	**	**	**
	CIBL	1.16^C b^	2.83^A a^	2.37^B a^	2.51^AB a^								
	CKBU	0.58^C c^	1.98^A c^	1.55^B c^	1.99^A b^								
	CKBL	2.31^A a^	2.57^A b^	1.45^B c^	1.72^B b^								

*^1^CI, Caragana intermedia; CK, Caragana korshinskii; BU, budding stage**;** BL, blooming stage.*

*^2^RB, 5% rice bran; RB + LP, 5% rice bran + commercial inoculant strain Lactobacillus plantarum; RB + L694, 5% rice bran + strain Lactobacillus 694.*

*^3^SEM, standard error of the means.*

*^4^V, varieties; G, growth stage; A, additives; V × G, interaction between varieties and growth stage; V × A, interaction between varieties and additives; G × A, interaction between growth stage and additives; V × G × A, interaction between varieties, growth stage, and additives.*

^5^NS, no significant, *p < 0.05; **p < 0.01.

*^6^DM, dry matter; CP, crude protein; aNDFom, neutral detergent fiber; ADFom, acid detergent fiber; ADL, acid detergent lignin; WSC, water-soluble carbohydrates.*

*^A–C^Means of inoculation treatments within a row with different superscripts differ (p < 0.05).*

*^a–c^Means of variety and growth stage within a column with different superscripts differ (p < 0.05).*

### *In vitro* Degradability of *Caragana* Silage

As shown in Table 2, the IVDMD was affected by varieties, growth stage, additives, and interaction between their pairwise combinations (*p* < 0.01). Compared with the control group, IVDMD content in additive treatments was significantly increased (*p* < 0.05). CI had higher IVDMD than CK (*p* < 0.05), while the budding stage had higher IVDMD than the blooming stage (*p* < 0.05). IVNDFD was mainly affected by the interaction of growth stage and additives (*p* < 0.05), added RB + L694 in *Caragana* Silage could significantly reduce the IVNDFD of CIBL, CKBU, and CKBL, and CK had higher IVNDFD (*p* < 0.05) than CK.

## Discussion

### Chemical Composition of Fresh *Caragana* and Silage

The fresh *Caragana* had high compositions of NDF and ADF. Bai et al. (2016) reported that high CP and ash compositions are detected in Ordos *C. intermedia* (19.86 and 8.24%, respectively), but aNDF and ADF are different to this report and it is likely due to the difference in location, varieties, clipping time, and years of cultivation. It is confirmed that the nutritional value is changed in the different growth stages of *Caragana* (Chen et al., 2010). As shown in Table 4, the aNDF, ADF, ADL compositions of CIBL and CKBU were lower than CIBU and CKBL, which revealed that varieties and growth stage had an interaction on fiber contents of *Caragana*. The aNDF, ADF, ADL, and WSC compositions of *C. intermedia* were significantly lower than that of CK. The *Caragana* shrub generally contains higher CP, because of nitrogen fixation with rhizobia of legume (Ji et al., 2015). *Caragana* at the blooming growth stage contained higher WSC and CP contents than the budding growth stage, due to the accumulation of nutrients during growth process (Xie et al., 2018). Therefore, based on the above analysis, the suitable cutting time for the two varieties is the flowering period.

Low fiber contents appeared in additive groups relative to control. We attributed that the lower aNDF, ADF, and ADL content in the silage inoculated with LAB may result from fibrinolytic enzyme production by other microorganisms during silage fermentation (He et al., 2020). The use of additives had no effect on crude protein content (*p* > 0.05), and both RB and RB + L694 treatments could enhance the compositions of WSC, while reducing the compositions of ADF, aNDF, and ADL of *Caragana* silage, which is due to the carbohydrate in rice bran, provides fermentation substrates for microorganisms, thus facilitating the preservation of silage nutrients. The CIBL and CKBL silages had higher CP content and lower aNDF and ADF contents than CIBU and CKBU (*p* < 0.05). Thus, the suitable cutting time for two varieties is the blooming stage according to the above analysis, and the *C. Intermedia* harvested in the blooming stage could result in better quality silage.

### Fermentation Characteristics of *Caragana* Silage

The decrease in pH is considered an important index to reflect the silage fermentation process, and the accumulation of NH_3_-N during the ensiling process is commonly recognized as the indicator of protein degradation (Nkosi et al., 2010). The dynamics of pH and NH_3_-N of *Caragana* silage indicate that varieties and growth stages, also rice bran and *Lactobacillus*, could influence the fermentation process.

The RB + L694 treatment exhibited lower pH and NH_3_-N than control, which indicated good fermentation. It is challenging to make high-quality silage for legumes, and single or combined bacterial inoculants have been used to improve the silage fermentation by accelerating the production of LA to inhibit the growth of undesirable bacteria and fungi, thereby reducing the nutrition loss (Cai et al., 1998; Eikmeyer et al., 2013; Bao et al., 2016; Ogunade et al., 2017; Muck et al., 2018). Treating with RB + L694 could increase the contents of LA and LA/AA and decrease pH and NH_3_-N contents compared with the control group. This suggested inoculation of commercial LP or L694 with 5% rice bran at ensiling *Caragana* could improve the fermentation quality of silage.

In the current experiments, silages prepared from the budding stage exhibited low fermentation quality. Due to the low WSC content of *Caragana* in the budding stage, the fermentation substrate was less and the fermentation was incomplete, as reflected by lower LA and higher pH. It has been proved that growth stage affects aerobic losses, which tended to increase as the plants aged (Weinberg et al., 2010). The aerobic stability of additive treatment was higher than control treatment. At the blooming stage, all treated silage had higher aerobic stability than the control, and *Lactobacillus* 694 had the best effect on particularly improving aerobic stability. Therefore, the CIBL had better fermentation quality, which could improve aerobic stability by plus rice bran and a selected strain L694.

### The Microbial Community of *Caragana* Silage

The high coverage value of each sample was about 0.99, indicating that most of sequencing was detected. High bacterial richness and diversity consisted in fresh *Caragana.* In our study, CK at BU and BL stages treated with RB + L694 had lower Shannon index and higher Simpson index than that of control, indicating the decrease in bacterial diversity and richness. This suggests that adding rice bran to CK silage in both growth stages can provide a basis for lactobacillus fermentation (Su et al., 2021), which can then grow rapidly under anaerobic conditions.

The fermentation quality of silage is highly dependent on the epiphytic microflora because ensiling is a bacterial-driven fermentation process. *Firmicutes* and *Proteobacteria* were the dominant microorganisms in legumes before ensiling (He et al., 2020). In our study, *Proteobacteria* were the dominant phylum in fresh material (57.24, 37.10, 62.12, and 54.50% for CIBU, CKBL, CIBU, and CKBL, respectively). *Proteobacteria* are gram-negative bacteria, which will compete with LAB to utilize WSC, resulting in the decreasing CP content and the increasing NH_3_-N content (Madigan, 2006). *Firmicutes* as gram-positive bacteria can degrade many macromolecular compounds, such as starch, protein, and cellulose (Song et al., 2015). After silage fermentation, the bacterial community mainly evolved into *Firmicutes*, and this may be caused by an increase in *Lactobacillus* and *Enterococcus*, both of which belong to *Firmicutes*. The present findings are consistent with that of Yuan et al. (2020), who proved that *Firmicutes* increased significantly in Napier grass silage as storage period was prolonged. After 60-days fermentation, *Firmicutes* were the dominant phyla and their number increased, while the number of *Proteobacteria* decreased, which was consistent with the report of Zhang et al. (2019). The decrease in aNDF, ADF, and NH_3_-N in silage might be related to this.

In order to further investigate the bacterial community during silage fermentation of *Caragana*, this experiment analyzed the changes in bacterial compositions at genus level. It showed that two growth stages could change the bacterial community of fresh material. The diversity and richness of bacteria decreased in CK silages treated with RB + L694, compared to those in the control silage.

*Lactobacillus* was the dominant genus in *Caragana* silage, which plays an important role in pH reduction at the later stage of ensiling (Cai et al., 1998). *Lactobacillus* abundance and fermentation quality in RB + L694 silage were higher than other silage because the exogenous *Lactobacillus* had a greater capacity to produce LA than epiphytic LAB of fresh material (Ali et al., 2020). As a kind of cumulative anaerobic bacteria mainly producing L (+)-LA, *Enterococcus* plays a positive role in improving the quality of *Caragana* silage. In this study, RB + LP treatment increased the number of *Enterococcus* in CIBU, CKBU, and CKBL. This indicated that the addition of LAB in *Caragana* silage increased the content of LA in silage and improved the fermentation quality, which was consistent with the research results of Tohno et al. (2013). Ensiling increased *Lactobacillus* and *Enterococcus* proportion in *Caragana* plant silage, which may contribute to the silage fermentation process to form an acidic environment so that most microorganisms were inhibited. This is consistent with the results reported by Cai et al. (2020). *Kosakonia* was the dominant microbe in the mulberry leaves and stylo silage and was pathogenic bacteria. The abundance of *Kosakonia* was 28.87, 20.36, 23.40, 4.58, and 9.79% in RB and RB + LP treatment of CIBU, control and RB treatment of CKBU, and RB + LP treatment of CKBU, respectively. The blooming stage of *Caragana* silage decreased *Kosakonia* abundance, indicating that undesirable microorganisms were reduced at the blooming compared with the budding growth stage. *Enterobacter* is facultative anaerobes, which is considered undesirable bacteria in silage, especially in silage with rich protein, because it can metabolize WSC and LA to produce other products, resulting in a nutrient loss in silage (Madigan, 2006). In CIBU, CKBU, and CIBL silages, *Enterobacter* was existed in untreated silage and treatments with RB silage, indicating that the LAB population did not inhibit the growth and propagation of undesirable microorganisms, and the addition of RB may promote the growth of *Enterobacter* (Keshri et al., 2019). As plant pathogens, *Pantoea* was also detected in fresh soybean and alfalfa. In our study, ensiling greatly decreases undesirable microorganisms (*Pantoea*). Thus, adding rice bran and inoculant strain *Lactobacillu*s gives rise to the increase in LAB proportion and inhibits the growth of harmful bacteria in *Caragana* silage.

### *In vitro* Degradability of *Caragana* Silage

The IVDMD at the blooming stage was higher than that in the budding stage, indicating that more DM was available in the blooming stage. The higher IVDMD of CI silage may be due to their lower fiber, because of the decomposition resistance of fiber content (Jung and Allen, 1995; Zhang et al., 2015). In this study, IVNDFD (48 h) decreased significantly with the maturation of CK (*p* < 0.05), which was similar to previous reports (Bai et al., 2016), and this may be due to the increase in ADF content with increasing maturity. The RB, RB + LP, and RB + L694 groups increased digestibility of silages, as reflected by the increasing IVDMD. Previous studies had suggested that LAB reduced DM loss in silage, and high IVDMD was detected in silage inoculated with LAB than that without LAB (Liu et al., 2016). In this study, RB + L694 group had greater IVDMD than other treatments, because RB + L694 had better inhibitive effect on DM loss.

## Conclusion

The forage growth stage has a greater impact on fermentation quality and nutritional value of silage. Growth stage had effects on chemical composition and *in vitro* digestibility, and the suitable cutting time for two *Caragana* species is the blooming stage. Rice bran and LAB inoculants increased WSC and LA concentrations of *Caragana* silage. *L. plantarum* 694, as a screening lactic acid bacterium, performed better than commercial *L. plantarum* on fermentation quality. In the blooming stage, RB + L694 treatments for two *Caragana* species could enhance the abundance of *Lactobacillus* and inhibit the growth of harmful bacteria such as *Enterobacter* in *Caragana* silage further improving *Caragana* silage fermentation. The best fermentation group was RB + L694 treatment of CIBL, and the group that ferments best was RB + LP and RB + L694 of CKBL. This study will provide the theoretical basis for the scientific and rational utilization of *Caragana* resources.

## Data Availability Statement

The sequencing data were submitted to the NCBI Sequence Read Archive database (accession number: PRJNA815495).

## Author Contributions

GZ and YC designed the experiments. HZ and HFZ ensiled the *Caragana* and collected the samples. JY and HZ analyzed the data and wrote the manuscript. YX and YC helped supervise the manuscript writing. All authors read and approved the final manuscript.

## Conflict of Interest

The authors declare that the research was conducted in the absence of any commercial or financial relationships that could be construed as a potential conflict of interest.

## Publisher’s Note

All claims expressed in this article are solely those of the authors and do not necessarily represent those of their affiliated organizations, or those of the publisher, the editors and the reviewers. Any product that may be evaluated in this article, or claim that may be made by its manufacturer, is not guaranteed or endorsed by the publisher.
